# ‘They said, let’s teach you how you are going to care for the child at home…’: caregivers’ and healthcare worker’s perceptions and experiences of post-discharge preterm care in eastern Uganda

**DOI:** 10.1186/s12913-022-08894-3

**Published:** 2022-12-14

**Authors:** Holly Lyne, Kathy Burgoine, Collin Ogara, James Ditai, Melissa Gladstone

**Affiliations:** 1 Department of International Child Health, Liverpool School of Tropical Medicine, Pembroke Place, Liverpool, UK; 2 Neonatal Unit, Department of Paediatrics and Child Health, Mbale Regional Referral Hospital, PO Box 921, Mbale, Uganda; 3 Department of Women and Children’s Health, Institute of Translational Medicine, University of Liverpool, Alder Hey Children’s NHS Foundation Trust, Eaton Road, Liverpool, UK; 4 Mbale Clinical Research Institute, MCRI, Mbale, Uganda; 5 Sanyu Africa Research Institute, SAfRI, Mbale, Uganda; 6 Busitema University Faculty of Health Sciences, Mbale, Uganda

**Keywords:** Preterm, Low-birthweight, Community, Post-discharge, Neonate, Africa, Low-income, Home care, Healthcare workers

## Abstract

**Background:**

Complications of prematurity are the leading cause of neonatal mortality, and the majority of these deaths occur in low and middle-income countries. Research in these settings has focused on improved outcomes for preterm infants in hospital settings, however, research into the continuation of preterm care in the home after discharge from a neonatal unit is limited. This study examines the experiences and perceptions of caregivers of preterm infants during the initial weeks following discharge from a neonatal unit in Uganda, and the views of healthcare workers (HCWs) on the ability of caregivers to cope.

**Methods:**

This qualitative study used multiple data collection approaches, namely focus group discussions (FGDs), in-depth interviews (IDIs), field observations, and case studies to explore the perceptions and experiences of providing care to preterm infants post-discharge from a neonatal unit in eastern Uganda from the perspectives of caregivers and HCWs.

**Results:**

We recruited 39 participants with a total of 35 separate sessions including 18 IDIs (12 caregivers and 6 HCWs), 3 FGDs (17 caregivers), and 4 case studies (14 separate IDIs over 5 weeks after discharge, three mothers, and one grandmother). IDIs and FGDs took place at the Mbale Regional Referral Hospital or in participants’ homes.

Key themes emerged; preparation for continuing care in the home, psychosocial challenges to providing preterm care in the home, barriers to continuing preterm care in the home, and suggestions for improvement of preterm care in the home. Caregivers had good knowledge and awareness about different aspects of preterm care. Following discharge, caregivers struggled to maintain quality care due to loss of continuous support from the neonatal team, feelings of anxiety and isolation, financial issues, and home responsibilities.

**Conclusion:**

This study highlights multiple challenges to continuing preterm care in this Ugandan setting. Improved training and education for caregivers, especially in neonatal resuscitation, enhanced and continued support of the caregiver and infant in the home, and increased community involvement following discharge may all be key solutions. These findings are fundamental to improving care in the home for preterm infants in eastern Uganda and similar settings.

**Supplementary Information:**

The online version contains supplementary material available at 10.1186/s12913-022-08894-3.

## Background

The leading cause of neonatal mortality is complications from preterm birth (WHO, 2016a). It is estimated that globally, 15 million babies are born preterm every year, with two-thirds of these born in low- and middle-income countries (LMICs) [1]. Although previously it has been estimated that only 50% of babies born at 32 weeks of gestation in LMICs survived [1, 2], more preterm babies than ever before are surviving to discharge [3–5]. When discharged home, parents of preterm infants are still required to provide additional care in the community in order to prevent infection and hypothermia, and to support growth and development,. There are a number of proven interventions that neonatal healthcare workers expect parents of preterm infants to provide after discharge. These include; kangaroo care, exclusive breastfeeding, and/or expression and administration of breast milk to their infant and infection prevention [5]. Kangaroo care significantly improves neonatal survival rates through prevention of hypothermia, facilitation of breastfeeding, and prevention of infection [6–8]. In preterm infants, breast milk improves growth as well as reducing the risk of developing sepsis, necrotizing enterocolitis, retinopathy, neurodevelopmental impairment [9–15]. Furthermore, preventing the transmission of infections through good hand hygiene is also an effective, low-cost means of infection control [16, 17].

To support these vulnerable preterm infants after discharge, the ability of parents to continue the provision of these key, life-saving interventions at home is vital. Despite this, it is unclear how well these high-risk infants are managed after they are discharged home. Data from Uganda show that almost one-third of very low birth weight infants die in the first 8 months after discharge from a neonatal intensive care unit, with two-thirds of these deaths occurring in the first month after discharge [18]. Few studies, particularly from LICs, have investigated how caregivers of preterm infants think and feel once they are finally discharged home and how capable they feel to continue with the recommendations and care they were taught in the hospital [4, 19, 20]. Some studies from HICs have demonstrated that many parents have feelings of insecurity, fear and anxiety after discharge, with these feelings continuing for a few months [21–24]. The lack of preparation for discharge has been identified as a key source of this fear. These feelings are often mixed with relief that their preterm infants are considered healthy enough to be discharged and the relief to be leaving the stressful environment of the neonatal unit [22, 23]. Caregivers also report feeling ‘isolated’ following discharge, often due to feeling an overwhelming need to provide constant surveillance of their infant and a fear of contracting infections, both of which inhibit their chances of socialisation with other community members [21, 22]. Some fathers are reluctant to be involved in the care of the preterm infant, leaving the mother alone at home and adding to mother’s feelings of isolation [22].

Some studies from both HICs and LMICs have demonstrated how responsibilities in the home, including housework, cleaning and care of siblings provide a considerable challenge for caregivers [19, 20, 25–27]. There are often competing demands for a mother’s time including having to spend much of the day working in the fields [28]. This results in a reduced amount of time available for the care of their preterm infant and can contribute to feelings of exhaustion [24, 26].

It is possible for these preterm interventions to be taught to parents during the inpatient admission and for them to continue them after discharge. Some studies have highlighted lower levels of parental education to have a negative impact on the continuation of preterm care in the home [23]. Other studies have reported inadequate training of the caregivers in appropriate feeding and kangaroo care before discharge, as well as a lack of knowledge of the caregivers of the benefits of continuing these practices at home to be key factors [23, 25, 26].

Most research on the barriers and facilitators to providing care to preterm infants post-discharge has been confined to HICs [22, 29–33]. In LMICs, research has focused on the initiation of care in the community for moderate to late preterm infants [20, 26, 34–36]. There is limited qualitative research in sub-Saharan Africa (SSA) on the experiences and journeys of parents once their preterm infant is discharged from a neonatal unit. Given that in-patient survival rates of increasingly preterm infants in LMICs are improving, particularly in SSA, understanding how best to improve the care of these high-risk infants post-discharge is vital. It is evident that, caregivers need to have the knowledge and the ability to meet the basic needs of their infants to prevent them from becoming unwell, and to promote good nutrition and development [32]. It is also clear that many caregivers in LMICs are unable to continue an adequate level of care for preterm babies in the community. This qualitative study examines the perceptions and experiences of mothers, caregivers, and healthcare workers on the journey following discharge of preterm infants from a neonatal unit in eastern Uganda. This study has been conducted to identify perceived barriers and potential facilitators to improving post-discharge care of preterm infants in the community.

## Methods

Three different methods of data collection, IDIs, FGDs and case-studies, were used in this study to collect data from carers and key-informants. These different methods of data collection and sources of data are necessary for triangulation and were chosen to ensure a thorough exploration of key themes [37]. We used purposive, non-random sampling to identify the most suitable participants for the IDIs, FGDs and case-studies. Although this may have introduced bias, it was necessary to select health workers and family members that had experience of caring for a preterm infant. Individual characteristics also provided the basis of selection, ensuring that the research sample reflected the diversity of people living in the community including participants from different tribes, different roles and different ages. For example, mothers, grandmothers and fathers of different ages were selected. This ensured that there was appropriate diversity between participants. The suitability of key-Informants was determined, and selection made, following the advice of the neonatal lead at the MRRH. This ensured that healthcare workers who had a broader and more extensive experience of caring from preterm infants were included, as well as a range of different cadres of healthcare workers including nurses and clinicians.

Data collection took place from May to July 2017. We recruited 39 participants in the overall study (a total of 35 separate sessions including 18 IDIs (12 caregivers and six HCWs), 3 FGDs (17 caregivers (mothers, grandmothers, and fathers)), and a further four case studies (14 separate IDIs) were carried out with caregivers (three mothers and one grandmother), followed up for 4–5 weeks after discharge. IDIs and FGDs took place at MRRH or in participants’ homes (Table 1). Further demographic details are provided in supplementary material (Supplement 2).

**Table 1:** Numbers and roles of the participants in the IDIs, FGDs and case studies

	Mother	Grandmother	Father	Aunt	HCW	Total
IDIs with Caregivers	8	3	1	-	-	**12**
IDIs with HCWs	-	-	-	-	6	**6**
FGD1	3	-	-	1	-	**4**
FGD2	4	1	1	-	-	**7**
FGD3	3	2	-	1	-	**6**
Case-Studies	3 (11 IDIs)	1 (3 IDIs)	-	-	-	**4**
**Total**	**24**	**5**	**2**	**2**	6	**39**

### Setting

This study was conducted in Mbale Regional Referral Hospital (MRRH), a public hospital that serves a population of 4.5 million people in eastern Uganda. MRRH has nearly 10,000 deliveries a year and also receives neonatal referrals from surrounding health facilities [38]. MRRH has a dedicated level-2 neonatal unit (NNU) that can provide intravenous fluids and medications, nasal oxygen, and bubble continuous positive airway pressure with up to 2,500 neonatal admissions per year, including almost 800 preterm neonates. In addition to one-on-one explanations to mothers, education on cord care, kangaroo care, hand hygiene, and temperature monitoring is delivered daily on the NNU by a healthcare assistant.

MRRH is a regional centre of excellence for neonatal care in Uganda and has achieved a 57% reduction in preterm in-patient mortality over the previous seven years [3, 39]. The neonatal lead and co-author (KB) had observed poor follow-up attendance and poor compliance with preterm care practices after discharge from the NNU. The need to improve understanding of the challenges that existed in the community after discharge as a priority in improving preterm outcomes had been identified.

### Individual interviews

Caregivers were identified from MRRH-NNU or the weekly neonatal follow-up clinic of recently discharged patients. All caregivers (mother, grandmother (“jaja”) or father) of any preterm infants who had recently been admitted to NNU were eligible to take part. There were no exclusion criteria. The suitability of key informants was determined, and selection was made following the advice of the neonatal lead and based on those with the most experience on MRRH-NNU. Potential participants were provided with verbal information as well as an information leaflet either in English or Luganda. Informed written consent was sought from all participants, those who were illiterate gave a thumbprint to sign their consent form. Case-study participants were re-consented at each interview. There was no remuneration for involvement. Unique letters were assigned as identifiers to each participant and used throughout in place of the participant’s name to ensure that the identity of each participant would not be recognised by anyone outside the research team. All data used was kept securely on a password-protected laptop.

### Focus groups

Mixed groups were used for the FGDs to ensure diversity as it was difficult to recruit enough men to have a group on their own. The FGDs were moderated by the research assistant to ensure that all the participants gave responses. We conducted IDIs with HCWs who were currently working, or who had recently worked, in MRRH-NNU.

### Case studies

We conducted four case studies with caregivers to allow for the exploration of individuals’ experiences within their real-life context and a holistic analysis of the case. We followed these caregivers for a period of four to five weeks after discharge every one or two weeks.

### Data collection

All IDIs with the HCWs were conducted in English by the primary researcher (HL), a white British young female Masters student. A research assistant, who was a young Ugandan female midwife (Diploma) with three years of experience in qualitative research and who spoke both English and Luganda, conducted all other IDIs and FGDs in either English or Luganda, as appropriate. Neither the primary researcher nor the research assistant were known to the participants prior to the IDIs/FGDs. IDIs lasted between 30 and 60 min and FGDs lasted between one and two hours.

IDIs were conducted either in a quiet room at MRRH or at the participant’s home enabling good rapport between interviewer and participant [40]. Interviews were structured using topic guides (Supplement 1), and depending on participants’ answers, the interviewer questioned further to gain a complete understanding of the participant’s perspective [40]. FGDs involving mothers and caregivers were structured using topic guides (Supplement 1) based on key themes that had emerged during the IDIs [40]. Topic guides were reviewed by the research team, piloted before interviewing began, and were iteratively adapted throughout.

All IDIs and FGDs were recorded on a digital voice recorder. To ensure consistency, interviews not undertaken in English were carried out by the same research assistant, who was familiar with the topic guides. Audio files were then transcribed and translated. All transcription of non-English interviews was carried out by the same transcriber. A reflective diary and field observation notes were taken by HL as part of the research work to support the analysis of themes and ideas.

### Analysis

Analysis was iterative, taking place during data collection and continuing afterwards. *Data analysis was carried out using the framework approach as this method is systematic and ensures rigour and transparency* [41]. All interviews were transcribed and discussed with the research team to also enable for adaptation of the topic guides. Analysis and coding began deductively with reference to the topic guide, identifying emerging themes in the transcripts. Analysis became more inductive as transcripts were then coded to finally establish a coding framework. To ensure a clear process of coding, initial transcripts were independently coded by three members of the research team. This meant that different viewpoints were considered, and no one point of view dominated the overall analysis [41]. After this, coding was completed by HL. Data analysis then continued using the framework approach as this method is systematic and ensures rigour and transparency [41]. NVIVO software (version 11) was used to chart the interview data into the framework. Throughout this process, the framework was continuously revised, and further themes were identified. The data from the FGDs and the IDIs with both the HCWs and caregivers were combined thematically. Data from the case studies was reported separately as these data focused on the challenges that emerged over a period of time after discharge. Data was then interpreted and explanations and reasons for the emergence of phenomena were sought. To begin with, the sample size was flexible and was guided by the concept of ‘saturation’. When additional data from further participants resulted in repetition and no new additional ideas being gathered, ‘saturation’ was assumed and no further IDIs or FGDs were held.

### Quality control

During this study, every attempt was made to ensure the credibility, dependability and confirmability of the research [37]. Different methods of data collection and sources of information ensured that results are comprehensive to allow patterns of convergence between sources to develop [37]. All members of the research team had experience in this particular medical setting and were known to participants. They were therefore able to build trust and rapport with the participants and this minimised the Hawthorne Effect [42]. The primary researcher received formal training from Liverpool School of Tropical Medicine (LSTM) in qualitative data collection and methods in order to ensure the quality of data collected [43] All research assistants also received local training in these methods and had previous experience. Participant checks throughout the data collection period were undertaken. If ambiguous answers were given, interviewers questioned participants to clarify the participant’s views. Following interviews, data was analysed and discussed within the research team. Any ambiguity in participant responses were then clarified, either by questioning the same participant, or where that was not possible, by conducting further interviews with different participants [44].

## Results

**Fig. 1:**
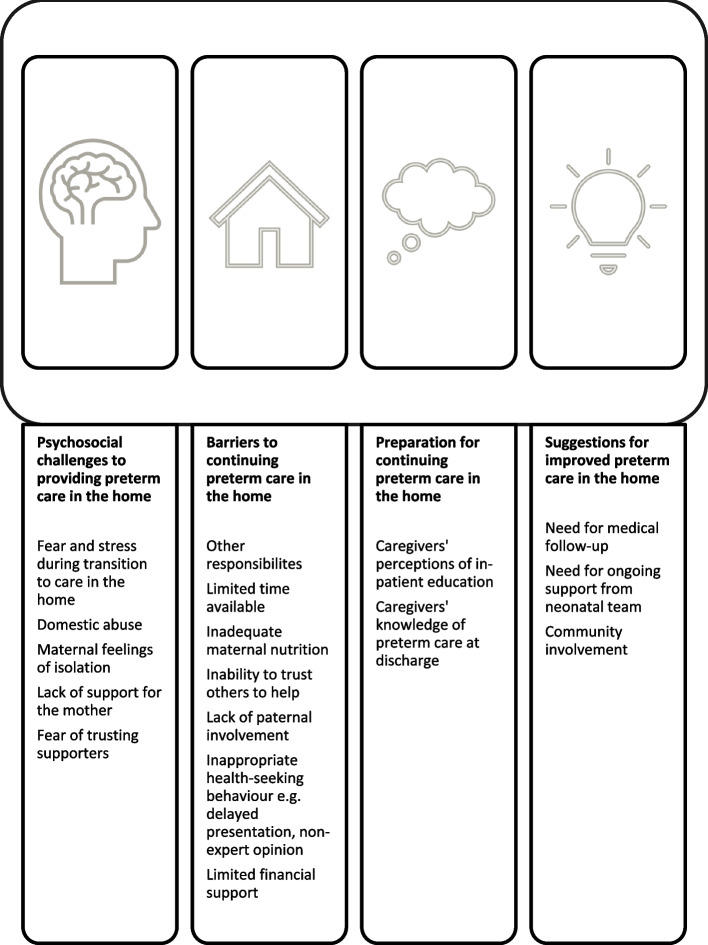
Diagram summarising the themes that emerged in IDIs, and FGDs, with caregivers and HCWs about providing care to preterm infants in the home after discharge from the neonatal unit

We provide our results thematically for both FGDs and IDIs combined. Four main themes arose from the analysis including preparation for continuing care in the home, psychosocial challenges to providing preterm care in the home, barriers to continuing preterm care in the home, and suggestions for improvement of preterm care in the home. Figure 1 summarises the key findings under each theme. The experiences of continuing preterm care from home from the four case studies (3 mothers, 1 grandmother) are presented as two vignettes. Each participant was interviewed three to four times over 5 weeks post-discharge. Lastly, potential solutions for the improvement of preterm care in the community are presented.

### Theme 1 – Preparation for continuing preterm care in the home

Under the main theme of preparation for continuing preterm care in the home, there were two key sub-themes as shown in Fig. 1; caregivers’ perceptions of in-patient education provided and caregivers’ knowledge of preterm care at discharge. The key perceptions and understanding of preterm care practices by caregivers and HCWs are shown in Table 2.

**Table 2:** A summary of the views and knowledge of caregivers and healthcare workers (HCWs) regarding preterm care practices

	Caregivers	HCWs
**Kangaroo Care**	*‘We make kangaroo for the child to get the same warmth as she was in the womb… you carry and put him here, you make sure that the neck balances, the head has to be up a bit, his stomach to be on your stomach … and you get a piece of cloth and tie on his back’ – FGD.M.11*	*‘We tie the baby on the mother’s chest and then the mother will be generating the heat for the baby, and therefore preventing the hypothermia’ – IDI.HCW1.1*
**Feeding**	*‘The breastfeeds, they told me after every two hours to be feeding… and also with the spoon’ – IDI.M.7* *‘I have to give only milk until he makes nine months’ – FGD.A.2*	*‘They know their times of breastfeeding … they are encouraged to feed the children on time… we also tell them to give nothing else but breast milk… we advise that they add the milk by spoon’ – IDI.HCW1.4*
**Controlling Visitors**	*‘When we go back home … I keep back my child in the bedroom and do not allow people to see her or handle her’ – FGD.GM.2*	*‘They must limit the visitors when they are at home… there should be minimal handling of these preterms, not everyone is supposed to visit the premature’ – IDI.HCW1.2*
**Hand Hygiene**	*‘You have to wash your hands well before handling the child, don’t give milk when you’re dirty’ – FGD.M.10*	*‘They know that they have to wash hands before they breastfeed and before they start expressing milk’ – IDI.HCW1.4*
**Maintaining Temperature**	*We just touch on the baby and when we feel he is too hot or otherwise too cold. So, they told us to maintain that temperature by covering the baby using heavy clothes of the baby – IDI.F.1*	*They just touch, and they know this baby, the temperature is very low… they can tell. But we also empower those who can afford to buy thermometers – IDI.HCW1.4*

#### Subtheme: perceptions of in-patient education provided

Approximately half the caregivers involved in IDIs stated that they felt prepared to return to the home environment on discharge and felt well-prepared to take on full responsibility for the care of their preterm infants. The remaining caregivers interviewed did not share the above view, considering that on the day of discharge they were not prepared; “that *whole first day, my baby was in my hands, I was just looking at him and I was so scared’ – IDI. 1.* Reasons included experiencing difficulties in different aspects of caring, particularly kangaroo care and feeding; *‘I had not mastered how to feed on spoon’* – FGD.F.1; ‘*for me when they were going to discharge me… they never taught me the care and I am just learning it from home now’ – FGD.M.3.*

HCWs considered that daily education of caregivers on the NNU was an efficient and effective method of health education; “*I teach the people on the ward. … when there are many there… teach them when they are in a group. So that many are able to learn together. On the ward you know this side is for the preterm baby, …so you can find all of them, and they will learn’ – IDI.HCW2.1.* There was a perception that staff on the NNU were incredibly busy and didn’t have the necessary time to devote to individual patients. HCWs from NNU agreed they were over-worked and frequently pushed for time, and that health education suffered as a result; *‘I think sometimes we are so overwhelmed with work that we don’t really have enough time, not only enough time but enough staff, to see to one-on-one …and talk to this mother fully. At discharge, we should sit with you and explain … you have to do this, do this, do this’ – IDI.HCW2.1*.

Caregivers felt that spending time on the ward caring for babies under the supervision of NNU staff was good preparation for continuing care in the home; ‘*They prepared me well … they said let’s teach you how you’re going to care for the children at home’ – IDI.GM.2.* Similar views were held by the HCWs; *‘I think because the longer they spend in hospital, they kind of get exposed to the environment that we teach them here… they must wash their hands, they don’t allow visitors’. – IDI.HCW2.1.* HCWs agreed that caregivers were ‘*encouraged to just continue what they have been doing there’ – IDI.HCW1.4*. There was a strong belief, among HCWs and caregivers, that talking through a plan of care on a one-to-one basis and questioning caregivers to determine what they did and did not know, was helpful preparation for discharge; *‘I just put it into practice everything I was used to when I was here on the ward, so it didn’t give me so hard time’ – IDI.F.1*.

While the mother is recovering from delivery, family members such as aunts and grandmothers are often the primary caregivers in NNU. These caregivers are often not involved in the infant’s care post discharge, and it was invariably the mother who took on the role of primary caregiver following discharge. The mother therefore had gaps in her knowledge, as information regarding care had not been adequately relayed from the caregiver; *‘When they discharged her, I was not there, … the person who was taking care of the baby never told me anything’ – FGD.M.3; ‘Sometimes we imagine they know because they have stayed on the ward for a long time, yet, maybe we gave the information to the wrong person’ – IDI.HCW1.4; ‘you find the mother is away from the baby, and all this information, this continuous health education you are conducting, you are actually giving to the wrong caretaker, then… this mother has no idea of how the baby is supposed to be cared for.’ – IDI.HCW2.1.*

#### Subtheme; caregivers’ knowledge of preterm care at discharge

Overall, caregivers reported having a good level of knowledge regarding all aspects of care for their preterm infants. In each of the areas questioned, feeding, medications, kangaroo care and infection prevention, the majority of caregivers demonstrated knowledge in line with what HCWs reported having taught. Nine of the twelve caregivers believed that they had sufficient knowledge to provide good quality care in the home.

HCWs also stressed the importance of caregivers attending follow-up appointments, stressing that caregivers are encouraged to return immediately to MRRH-NNU if they had health concerns about their baby. All caregivers understood this, saying that they would not take their preterm to any other healthcare facility to receive care.

Caregivers had little knowledge of how to carry out neonatal resuscitation, some caregivers mentioned being extremely concerned that their babies might stop breathing as they had witnessed this happening to other babies on NNU. Caregivers interviewed were asked what they would do if their baby stopped breathing. Only two suggested techniques they would try in such a situation, which included *‘rubbing their chest’* or ‘*blowing over their face’* – IDI.M.6. The only other response was that they would rush back to the hospital.

### Theme 2 – Psychosocial challenges to providing preterm care in the home

The second main theme was psychosocial challenges to providing preterm care in the home, under which there were four key sub-themes as shown in Fig. 1; fear and stress during transition to care in the home, domestic abuse, isolation and support for the mother.

#### Subtheme: fear and stress during transition to care in the home

A common concern during the first days at home was experiencing anxiety and fear that the baby would become unwell and that caregivers wouldn’t know what to do, or they *‘may not notice the problem’ –* IDI.GM.1. Caregivers missed the reassurance of having HCWs to rely on for advice and felt that the care of their babies would suffer in the home as a result. On the ward, staff reminded caregivers when to feed, and when to practice kangaroo care; *‘on the ward, in case there is something you don’t know, you can easily ask’ – IDI.M.3; ‘when we were there, the nurses could tell you that ‘no you have tied the baby poorly, put it in this position’… but when you are there at home you don’t know whether the baby is now in good position’ – IDI.F.1.*

However, other caregivers reported relief at being away from the busy hospital ward and reassured that their babies were considered healthy enough for discharge; *‘Being in the hospital is so hectic… I have nowhere to rest, and I sleep on the cement, … at home it is better. We are free, and I can give him the care which is needed.’ – IDI2905.*

#### Subtheme; domestic violence

Domestic issues included arguments, domestic violence and drink related issues. Caregivers awaiting discharge worried that ‘*people at home quarrel a lot’ – FGD1006*. None of the caregivers referred to intimate partner violence or domestic violence (IPV/DV). However, DV/IPV was a common concern among the HCWs interviewed and it is possible that many women might not have been aware that what they suffered was IPV/DV. Fights and arguments with family members, especially between parents, were discussed at length. Finally, issues of drug use, the partner’s excessive alcohol consumption and its impact on the caregiver was mentioned by one caregiver; *‘his father doesn’t handle him, he leaves early morning and he comes back when drunk’ – IDI.M.3* and one HCW; *‘their husbands have abandoned them, there is all kinds of domestic violence at home… so their mother is all stressed…and the conditions in home are not very good for her to care for her child’ – IDI.HCW2.1*.

#### Subtheme; maternal feelings of isolation

Caregivers reported that, on returning to the home, the support they had received during their time in hospital from friends and other family members disappeared, making the move from hospital to the home environment more difficult. *‘At first…, I was with my sister and my husband, but after one day, my husband had to go … to look for some money, then my sister had to go back home… so they left me alone with the two children… it became a very big challenge.’ – IDI.M.5*.

Caregivers reported spending entire days alone, often feeling unable to leave the baby’s room; *‘I can’t do any activity, because I cannot bring the baby out… I have to be in the bedroom,‘ – IDI.F.1*. Caregivers worried about the risk of infection outside the small and protected environment, which they considered infection-free: *‘because its I who cares for the child, I don’t move or work, I am just at home’ - IDI.M.4.* One HCW expressed the view that mothers should stay within their compound, close to the baby during early days following discharge; *‘I am going to tell the mother to be sitting inside the house with her child and not allow her to move with the child’ – FGD.A.2.*

#### Subtheme; lack of support for the mother and fear of trusting supporters

In six IDIs mothers expressed the view that no one else, including their husbands, was capable of providing adequate care, and therefore they preferred to take on sole responsibility. In most situations, mothers considered themselves the only person capable of carrying out kangaroo care. Even where support was offered, mothers would not accept it: *‘I had my sister, but I could not trust her much, because I wanted to keep the baby myself -* IDI.M.6.

Among HCWs, views were divided. One viewed sole care by the mother as positive; *‘…if there is a single attendant, and just the mother cares for that baby, there is limited infection; - IDI.HCW1.1*. However, another advocated accepting help and support from partners or family members, believing that this would result in better care for the baby, such as performing kangaroo care for longer periods of time; *‘there are relatives supporting, … and they can do kangaroo very well’ – IDI.HCW1.3*. Another cultural belief that emerged was that men should not be the ones to carry babies around, and that it is the woman’s role: *‘in Africa they think that men are not supposed to carry babies, so there is also that challenge’ – IDI.HCW1.2.*

All caregivers reported receiving some support from family members and friends. Although caregivers were often unwilling to accept support which directly involved care of the baby, they welcomed support in other ways, including moral support by visiting or phoning, and financial support; *‘they come, and they see (the mother), …and give 2000, 5000 shillings that helps’ –IDI.F.1.* Other support offered included taking over household chores such as washing, cooking and fetching water; *‘…like washing dishes, fetching water from the tap, even sometimes cooking. My niece can do domestic work’ - IDI.M.7.* This was seen as valuable, enabling caregivers to spend more time attending to their babies. *‘I get support from my husband, friends and relatives and they support me in whatever way I want, and I have more time. In case the clothes are dirty, or the baby has defecated, they rush to help’ – FGD.M.1*.

In IDIs, six of the twelve caregivers mentioned having either no support or insufficient support: *‘getting the support to help you care for the child is not there…because my husband is not there, and the others go to school’ – IDI.M.6*. Several caregivers mentioned that their ability to care was badly affected by friends and relatives sharing negative opinions with them about their situation. Caregivers found this demoralising: *‘others talk from behind saying that the child is going to die, and I am wasting my time’ – IDI.M.7.*

### Theme 3 - Barriers to providing preterm care at home

Under the theme of barriers to providing preterm care at home, six key sub-themes were identified as shown in Fig. 1; barriers to continuing kangaroo care, inadequate breastmilk production, infection prevention, health-seeking behaviour, other responsibilities and limited financial support. These barriers are summarized in Table 3.

**Table 3:** Summary of the views of caregivers and healthcare workers (HCWs) regarding the home environments of caregivers

	**Caregivers**	**HCWs**
**Weather**	*‘Sometimes it could rain …Then sometimes in the morning it was very cold…So it was a challenge to me.’ – IDI.M.5*	*‘The only challenge is, er, those you can’t control, you know like its rainy season’ – IDI.HCW2.1*
**Neighbourhood**	*It is not so clean, and it has a high population. Whenever I go out, many children gather, and they come to touch him, and it doesn’t make me happy.‘ – IDI.M.6*	‘I*n the environment where she stays there is so many people so whenever she takes the baby out, they say everyone comes around.’ –* IDI.HCW1.1
**Mosquitos**	*‘… and mosquitos are very many in the area’ – IDI.M.6*	*‘At least your home is ok, because you will keep your baby in the bedroom, nobody will come there anyhow. That’s the advantage of it.’ - IDI.HCW3.1*
**Visitors**	*‘The challenge there are some stubborn visitors who will just forcefully come to see them.’ – IDI.M.5*	
**Electricity**	‘*If there is no power, you can improvise using a charcoal stove, trying to cause some heat for the baby.’ - IDI.M.4*	

#### Subtheme; barriers to continuing kangaroo care

Caregivers reported being occupied with other tasks, which could not be performed with their baby attached to them; ‘I became busy and forgot kangaroo, like with the washing and tidying…’ – IDI.M.4. Caregivers said that the workload at home was an issue in providing care, when compared with time on the NNU when their sole responsibility was caring for their baby; *It wasn’t easy, I left here when tired and there is a lot of responsibilities at home compared to the hospital – IDI.M.*

Key-informants believed that the quality of care at home was significantly lower than that on NNU; *‘They struggle at home, because you know, these people are busy. Here, in the hospital they are not busy. They are just sitting, keeping the baby. But in the village, it is difficult, you cannot sit in one place because you are tying the baby in kangaroo, you must go and fetch water, go and do work. That is what I think the problem they have is. But here, they get the chance of tying their baby even 24 hours because they are not doing any work’. – IDI.HCW3.1*.

There were multiple misconceptions about kangaroo care. The almost unanimous opinion among caregivers was that it was impossible, or exceedingly difficult, to carry out other tasks whilst practising kangaroo care. Some caregivers stated they had attempted but failed to carry out basic household tasks, such as washing clothes, while their baby was tied to the front of them. *‘When you make kangaroo, there is no kind of work you do, you only sit there.’ – FGD.M.2*; *‘Actually there is no activity. I will be sleeping…, or I just stand. But there is not any other activity I can do while on kangaroo.’ – IDI.GM.1*. Many caregivers discontinued kangaroo care at home, believing that their babies were much happier when they weren’t being carried in this fashion; ‘*My baby will feel pain if I tie him, I think he would not be comfortable’– IDI.M.6.* Caregivers were also concerned that it might contribute to breathing difficulties; *‘the problem I have got is that when I make kangaroo and put the child on the chest… the child doesn’t breathe well’ – IDI.M.7.* Or it might be uncomfortable for the baby or even ‘*snap the baby in half’* – IDI.M.4; *If you’re not careful you can break your child, because they’re always slipping down, and very tiny’ – IDI.M.5*. HCWs did not share these concerns, recommending that caregivers moved around and attempted household tasks with their baby attached to them. Some were unaware of caregiver’s concerns, insisting that ‘T*hey love it – KI2605’.* These HCWs believed that caregivers continued kangaroo care in their homes to a high standard, facing no challenges; *‘They can do the kangaroo very well, because they know and have everything they need’. – IDI.HCW1.3*.

#### Subtheme; inadequate breastmilk production

Four mothers in IDIs complained of being unable to express sufficient milk and reported *‘pressing so hard until you feel pain’ – IDI.M.4*. HCWs maintained that the reason for problems with feeding were that mothers did not have sufficient food to feed themselves *‘so they are not able to make enough milk’* – IDI.HCW2.1. When mothers were asked how they dealt with poor milk supply, they explained that they just kept trying and eventually managed; *‘yes expressing, it became a little bit difficult for the two children. In that difficulty I persevered, I could do so’ – IDI.M.5.* One mother reported giving up and using formula milk. *‘I buy some other milk for him so that he can get satisfied. And actually, when I give him, he takes after every two hours I give that milk, he drinks and is happy and sleeps. But if I am only breastfeeding, the boy will just continue crying the whole night’ – IDI.M.6.* Additionally, one carer expressed the view that lack of lighting, due to lack of electricity, made expressing impossible during the night.

#### Subtheme; infection prevention

Two concerns were commonly raised concerning infection prevention, one being an inability to control the number of visitors entering the home. HCWs considered that, once back in the home environment, caregivers were better able to isolate there. However, many caregivers stated that, in the community, it proved far more difficult to isolate their babies: ‘‘*There is no way you can deny someone from seeing a new baby.*’ – IDI.M.6. Caregivers explained that visitors were extremely persistent, often entering the baby’s room against the carer’s will.

#### Subtheme; health-seeking behaviour

NNU was invariably cited as the first port-of-call for caregivers who had any concerns regarding the well-being of their infant. Caregivers would either return to NNU or communicate with staff via phone for advice. This support provided great reassurance. Faith also played a large role; caregivers took comfort in spiritual support through any difficult times following discharge. They maintained that this gave them strength to continue in their role as caregiver: *‘I always ask for God to protect us. For that support … there is nothing I do apart from praying’ – IDI.M.5.*

Five caregivers discussed transport difficulties as a result of poor finances, which meant they were unable to attend follow-up appointments; *‘I think one of (the barriers) is finances. Finances in terms of transporting themselves for these follow-up appointments, finances to feed themselves and to get some better things like the medication we ask them to give to the babies’– IDI.HCW2.1*.

#### Subtheme; other responsibilities

The most common barrier to providing effective care including kangaroo, feeding and infection prevention, was considered to be other responsibilities at home. Ten of the twelve caregivers spoke about this, and it was discussed in all three FGDs. Carer’s responsibilities involved household chores, such as cooking, washing clothes, cleaning, fetching water, digging and ‘*such things of the woman in the home’ (IDI29051)*. The only carer who didn’t mention this as a barrier was the only father involved in IDIs.

#### Subtheme; limited financial support

Six of the twelve caregivers, participating in IDIs, discussed difficulties arising from financial restrictions. It was a key theme discussed at length in all three FGDs. When asked whether money was an issue, only one carer considered the family to be financially stable. Yet when asked what helped caregivers to provide good quality care many responded ‘money’; *‘What has helped me most is money, without money it isn’t easy’*- *IDI.M.7*; *‘Financially, we are not all that stable. We struggle. So, the baby’s care is not that ok’ – IDI.M.6*.

Four of the six HCWs also identified money as an area of concern in the care of preterm babies. Caregivers made reference to requesting financial support from family members and friends. Lack of money meant an inability to buy necessary supplies. Insufficient food supplies for the mother affecting the mothers’ milk supply was mentioned by eight caregivers. Supplies that could not be funded included medicines, nappies, soap and thermometers. Frequently discussed was not being able to afford to buy thermometers, which caregivers had had access to the NNU. Without them it proved impossible to accurately monitor the baby’s temperature. This was an issue identified by key-informants, who discussed caregivers having to guess the baby’s temperature; *‘At home they may not buy a thermometer…that’s the challenge, they do not have the thermometer to know the baby is cold to tie the baby on kangaroo, they just suspect’. – IDI.HCW3.1*.

### Theme 4 - Suggestions for improvement of preterm care at home

Under the theme of suggestions for improvement of preterm care at home, two key sub-themes were identified as shown in Fig. 1; need for medical follow-up and community involvement.

#### Subtheme; need for medical follow-up

There was agreement among HCWs and caregivers over the need for follow-up appointments following discharge. Caregivers felt that a home visit from a hospital staff member would be beneficial in the early days following their discharge; *‘For me what I would like the staff to do …is to give you a visit, like on Sunday, to see how the baby is doing at home… and can help you care the other way round’. – IDI.F.1.* All HCWs referred to a lack of resources in the provision of follow-up care. Three HCWs believed that, in many cases, caregivers returning for follow-up appointments were not honest about how well they are coping at home. Wanting to be seen as compliant, caregivers are tempted to report that they are continuing care as instructed when *‘in actual sense maybe there is something they have not been doing right’* – IDI.HCW1.4. HCWs believed that in order to gain an accurate impression of care in the home, and to what extent support was needed, home-visits were necessary; *‘I think because we don’t know what goes on at home, it is important for us to go to visit them and their babies …, to see what exactly they are doing and not relying on what they say…and see if there is anything that we can ask them to improve. Maybe that will improve their care.’ – IDI.HCW1.4*.

Four caregivers in IDIs, and one in a FGD, mentioned phoning staff members at the hospital for advice following discharge. No caregivers mentioned the need for improvements in communication. However, HCWs suggested that better communication between caregivers and staff would enhance the care of preterm infants at home, indicating that there was a need for increased two-way communication. In their view, caregivers should be encouraged to contact the hospital more frequently with any concerns, and hospital staff should be available to check-up on the caregivers. *‘The barrier that I see… is no communication, with the hospital.’ – IDI.HCW1.3*; *‘What I feel is that …we should give them our contact before they go. In case of anything they can call back… so that we can advise these mothers on the phone what to do, …and likewise give (the caregivers number) to the nurse so she can inquire what is happening at home’ – IDI.HCW1.1*.

Both caregivers and HCWs mentioned that in order to improve the attendance at hospital follow-up appointments transport to and from the hospital should be provided; *‘…I wish for them to provide us with transport … that is the problem’ - IDI.GM.2*; *‘I would really love to have all these preterms come for the follow-up. … If I had the funding, I would try … to get them some kind of small money… for coming for follow-up appointments’. – IDI.HCW2.1*.

#### Subtheme; community involvement

Three HCWs emphasised the importance of increased community involvement. Suggestions included involving community and religious leaders in promoting education and training, together with the provision of community-level healthcare facilities, and further education via videos and radio programmes; *‘(Health facilities) in the community should also have knowledge on how to care for the babies. So, all the protocols we have, we should inform them’ – IDI.HCW1.1 ‘Interviewer: what could improve the care of preterm babies once they have been discharged? Participant: bringing the whole, the community on board. I would recommend outreaches. Go out and talk to communities. Go on the radio, and then keep on educating the community’ – IDI.HCW1.2*.

Caregivers suggested that group sessions would be beneficial in promoting continued education, so leading to higher quality of care; *‘Interviewer: what could improve your ability to care for your baby at home? Participant: …maybe it is through sharing, in classes with others the same. There you can enough knowledge and support, so you know what to do. – IDI.GM.1.*

### Case-studies: continuing preterm care from home

The main findings from the four case-studies are presented in two vignettes below (*the names have been changed*) and in Table 4.

#### Vignette 1: Sylvia, mother

Having spent three weeks in the NNU, Sylvia returned to her village with her preterm baby. She was relieved to leave the unit and felt she’d been taught to care for her baby well. She planned to do everything she’d learnt when back home.

A week after discharge Sylvia found she missed the support of hospital staff. Her mother, who had been present in the NNU, had returned to her own. Sylvia’s husband was working some distance away to provide for the family. Sylvia’s entire day revolved around caring for her baby and two older siblings. Unable to leave the house, she felt isolated. Receiving visitors concerned Sylvia as she believed they might increase her child’s risk of infection. On the NNU, she had practiced kangaroo care six to eight times a day, but did not continue with this, saying that it was impossible while trying to care for other children. She only continued kangaroo care when the baby felt cold. She experienced difficulty breastfeeding, was not producing enough milk, and found expressing milk painful. She was considering giving the baby cow’s milk.

Three weeks after discharge, Sylvia’s baby was breastfeeding well and had gained weight. Sylvia therefore felt able to leave her with a family member, which gave her time for shopping, and other household tasks, including dressmaking. Sylvia felt she needed to get on with life and therefore stopped all kangaroo care.

Five weeks after discharge, Sylvia’s husband had returned and, though he didn’t help with child-care, Sylvia felt more at ease. She’d been concerned when her baby had had a chest infection but explained that she prayed, and the baby’s health improved. Following her baby’s illness, she was even more concerned about visitors entering her home, but could not prevent them from doing so. Sylvia decided to attend a follow-up appointment, which she found encouraging. Sylvia was, on the whole, positive about her baby’s progress; ‘*The baby has improved in the weight… and I have improved my skills, so it gives me confidence.’*

#### Vignette 2: Doreen, grandmother

Doreen is the grandmother of a preterm baby. Doreen’s daughter is only fifteen years old, so Doreen took on the role of primary caregiver. She spent the first two weeks of the baby’s life on the NNU, where the baby had been exclusively spoon-fed. Doreen, has struggled to help her daughter with breastfeeding. She felt she needed support, but the father was not involved, and there was no help available from HCWs or family members.

Three weeks post discharge, Doreen’s daughter had managed to start breastfeeding, but the baby was still dependent on expressed milk given by spoon. Doreen had full responsibility for all other aspects of the baby’s care. She had missed two follow-up appointments.

Doreen was frightened, in week three, when the baby suddenly had serious difficulty breathing. Not knowing how to respond, she blew on the baby’s face, it didn’t work. She rushed the baby back to hospital and was readmitted to the NNU.

Five weeks following discharge, Doreen was coping a little better. Her daughter was breastfeeding successfully. She felt able to leave the baby with her daughter for short periods of time while she did household chores. Doreen was no longer practicing kangaroo care, explaining that the baby cried hysterically if tied to her chest while she attempted other tasks. The baby’s breathing was a constant concern for Doreen, but she knew she would not be able to return to the NNU; *‘… I tell them I don’t come because I need some money for transport.’*

**Table 4:** Summary of the main findings from case-studies at three consecutive points following discharge

	**1 week after discharge**	**2–3 Weeks After Discharge**	**4–5 Weeks After Discharge**
**Adjustment to Life at Home**	• Relief associated with returning home • Under-confidence in skills • Adequate knowledge for provision of care • Provision of good quality of care to babies • Isolation in the home • Some external support from family members	• Increased confidence in care provided • Reduced feeling of isolation in the home • Household activities commenced • Reduction in external support from family members	• Well-settled in the home • Confidence in skills • Contentment and pride • Generally, activities being undertaken outside the home
**Kangaroo care**	• Continuing in the home • Reduction in time devoted compared to on NNU • Lack of knowledge regarding appropriate frequency	• Further reduction in time devoted to it • Feeling it was no longer necessary	• Virtually ceased • Viewed it was no longer necessary • Fear that it might harm baby
**Feeding**	• Spoon-feeding expressed milk • Inadequate preparation for breastfeeding • Difficulties resulting	• Some difficulties associated with breast-feeding • Increased confidence in most cases • One report of resort to cow’s milk	• Increased confidence • Spoon-feeding ceased • Reports of baby breastfeeding well • Need for help with breastfeeding
**Health**	• General concerns regarding health of preterm • No specific concerns reported	• Continued improvement in baby’s health • Failure to attend follow-up clinics • Some serious health concerns • One readmission to NNU	• Improvements seen in general health and growth of babies • Pride associated with good health of babies
**Emerging Challenges**	• Withdrawal of hospital support following discharge • Little support from fathers • Financial issues	• No further challenges reported	• Concerns over inability to control babies’ exposure to visitors and possible transmission of infection

## Discussion

Our study presents data regarding the care of preterm babies at home following discharge from a neonatal unit in Uganda and looks at how caregivers were prepared for discharge, how they coped in the home setting after discharge and the challenges they faced.

The first key theme identified was preparation for continuing preterm care in the home. Overall caregivers felt well-prepared for discharge and many caregivers felt confident about continuing care post-discharge. They reported that they had received health education and opportunities to carry out different aspects of preterm care under the supervision of staff. Learning best practices in caring for preterm infants before discharge was seen as the key factor in enabling caregivers to provide high standards of care in the home. At this healthcare facility, the importance of education was recognised, and emphasis was placed on group teaching on the ward by a nursing assistant and supervised practice of different aspects of care before discharge. This proved effective, and compared to other settings, caregivers in this study appeared to have better knowledge about care on discharge from NNU [23, 25, 26]. However, as in Papua New Guinea and India, the education received by caregivers was limited by the fact that that staff were busy and did not always have sufficient time to provide education [45, 46] Limited human-resource in low-resource settings is a significant barrier to improving this and advocacy for additional roles in training and education is needed.

One key gap in the caregivers’ knowledge was highlighted: neonatal resuscitation. Many caregivers felt concerned that they would not know what to do if their baby stopped breathing. Research into caregivers’ awareness of neonatal resuscitation is limited. A qualitative study undertaken in a HIC similarly found that caregivers had little knowledge of preterm apnoea or bradycardia and were not able to say how they would respond to these situations. One literature review concluded that teaching neonatal resuscitation was beneficial in improving preterm mortality rates [47]. This, however, was based on limited evidence.

The second key theme identified in our study was the psychosocial challenges to providing preterm care in the home. While on NNU, caregivers benefit from 24-hour surveillance and have immediate access to support, advice and treatment when needed. Following discharge, this support is withdrawn as caregivers continue the care of their preterm baby alone. A qualitative study in Iran demonstrated that once in the home setting, caregivers’ confidence declined, as did their competence, due to the fact they had been dependent on staff at the unit [31]. Our research found that caregivers struggled at home where the NNU staff were no longer accessible to them. Similar findings have been reported in other settings [21, 22, 27]. Many caregivers stated that home visits during the initial weeks following discharge would have been desirable.

An important finding in our study was that caregivers felt isolated following discharge. In case studies, it was reported that only five weeks after discharge did caregivers feel comfortable leaving the home. An underlying reason for this was the carer’s belief that it was necessary to keep the baby away from other people to reduce the risk of infection. Similarly, in Tanzania, caregivers spent an average of 40 days isolated indoors with their preterm babies [35]. On NNU, the importance of restricting visitors to minimise transmission of infection is stressed. This may well contribute to caregivers’ isolation in the home as caregivers continue to adopt these measures in the home setting for several weeks post-discharge. Advice as to when restrictions can be eased may well contribute to caregivers accepting more support and feeling less isolated once back in the community.

Barriers to providing preterm care at home was the third and largest key theme we identified. Our study highlighted the fact that some caregivers commenced inappropriate practices on their own initiative, including supplementing breast milk with alternative feeds, for example, cows’ milk, and using charcoal stoves to keep their babies warm. Other research in similar African settings in Tanzania, Ghana and Uganda had similar findings [4, 35, 48]. In Tanzania and Ghana, caregivers often fed their babies sweetened water in place of breast milk [35, 48]. In Uganda, caregivers reportedly use charcoal stoves, or jerrycans of hot water, close to the baby’s bed to keep the baby warm [4, 49]. In India caregivers often wrapped their babies in blankets to maintain temperature, rather than practising kangaroo care [49]. Similarly, in this Ugandan setting caregivers wrapped their babies in blankets in an attempt to maintain their temperature, although kangaroo care had been taught as the most effective means of thermal care. Another, earlier finding in Tanzania indicates that caregivers would begin to bathe preterm babies too [35]. In our study, caregivers were unclear as to the appropriate time to start bathing at home. These are areas of care that could be clarified and discussed before discharge.

Another key finding from this study was that very often the mother was not the primary caregiver during the baby’s admission to NNU. Other family members took on this responsibility while mothers recovered. Mothers then took over the role of the primary carer in the home, having not received appropriate levels of training as information was not passed on. This finding has not been reported in previous studies and needs further exploration and intervention to minimise the negative impact.

Caregivers stressed the importance of support from family and friends following discharge, which was also the number one enabler in caregivers being able to provide good care. This was reported in a comprehensive, systematic review on the barriers to and enablers of care for preterm infants [50]. It has been found in other LICs, that support from partners was frequently non-existent due to work commitments and gender role stereotypes [51–53]. Similarly, in this study, it was frequently reported that care from family members decreased following discharge. It was only possible to interview two fathers as the majority were not available for interview. Interviewing a larger number of fathers would have provided more insight into how they regarded their role in the home. Research into the care given by fathers in the days following discharge is limited, however encouraging male involvement could improve the care of neonates in the community [54]. Research undertaken in HICs found that fathers’ involvement in care post-discharge was beneficial. Tackling perceptions concerning gender roles could therefore contribute significantly to improved support mechanisms for caregivers and involvement by fathers could enhance the quality of care.

Another finding in our study was that mothers were unwilling to accept help with the direct care of babies, their main reason being that they could trust no one else. This has been reported in other settings [25, 27]. Whereas caregivers in this study were not practising continuous kangaroo care, 99.5% of caregivers in Ghana, who accepted help from spouses and other family members, were still practising continuous kangaroo one week after discharge [55]. This stresses the importance of family members being involved in the care of preterm infants. It also highlights the need to explain to mothers that managing alone as sole carer for a preterm baby is a huge challenge and help, whenever offered, should be accepted in the best interests of their baby. Religion is also known to play a significant role in enabling caregivers in particular areas of the world. In Iran, the majority of caregivers put complete faith in religion, asking guidance from God as they continue to care for their preterm infants [27]. Partner involvement is also considered crucial to the successful continuation of kangaroo care in the community [34].

Although unwilling to accept direct support in the care of babies, caregivers mentioned requiring support regarding other household tasks. The need to divide time between caring for their preterm baby and other responsibilities such as cleaning, cooking and gardening. This was found to be a key challenge both in this research and across many other settings [4, 26, 53, 56]. In Papua New Guinea, where family members took over all other responsibilities in the house following discharge, caregivers reported practising kangaroo care for longer periods than in our study [45]. This provides evidence that continued support from others in sharing the workload is vital if preterm babies are to receive optimum care.

Caregivers experienced difficulties commencing breastfeeding or lacked the incentive to persevere with it. Preterm infants start breastfeeding at a later age than babies born at term, and in MRRH-NNU they were encouraged to express their milk and give it by nasogastric feeding tube or spoon. Although they are taught to hand express, most mothers did not get support with breastfeeding in NNU and were discharged into the community without having had to practice in this area. Most mothers began breastfeeding at home, without advice and support, and reported challenges such as experiencing pain and lacking sufficient milk. This contributed to under-confidence in their ability to breastfeed. This was also found to be the case in other settings [31, 57] Research indicates that nurses play an important role in providing support and reassurance to new mothers as they commence breastfeeding [57–59]. This demonstrates a need for continued close contact between mothers and HCWs following discharge.

An important finding in this study is that caregivers believed that it was impossible to undertake other tasks while their baby was in kangaroo. Caregivers strongly believed that any time spent practising kangaroo care would need to be spent sitting still and they saw this as time-wasting given the many other responsibilities they had. Being encouraged to move around more whilst practicing kangaroo care in NNU might encourage caregivers to do so at home and therefore increase the time spent in kangaroo.

Interviews with caregivers in Uganda and Ghana have identified that some mothers fear that kangaroo care might harm their baby as traditionally, mothers carry babies on their back [4, 26]. This results in caregivers practicing kangaroo care for shorter lengths of time at home than in neonatal units [23]. Furthermore, a Ugandan study identified low income, a heavy workload and a lack of decision-making power as key barriers to the practice of kangaroo care at home [34].

Caregivers in this study believed kangaroo care might harm their baby. This was in line with findings in other developing countries [26, 50] Caregivers feared that it was uncomfortable and detrimental to the baby’s breathing or that the baby might fall if carried around. Addressing these misconceptions could therefore increase caregivers’ willingness to continue practising at home. However, caregivers in this country experienced less pain and fatigue related to kangaroo care, and fewer cultural barriers than in many other settings [36, 55, 60, 61].

Financial considerations were responsible for caregivers’ failure to return to the hospital for follow-up appointments, or to NNU if their baby fell sick. This has also been reported in Malawi [62]. Transport problems were given as the main barrier to seeking healthcare advice following discharge. Caregivers and HCWs expressed a strong preference for returning to NNU at the referral hospital rather than attending community hospitals or healthcare facilities in the neighbourhood.

The last key theme identified was suggestions for improvement of preterm care at home. In HICs, good communication between caregivers and healthcare workers in neonatal units, as well as the need for continuing education and support in the community, have been highlighted as key enablers to comprehensive preterm care following discharge [21, 24, 25, 27, 63]. A Swedish study assessing the benefits of real-time video conferencing between a neonatal unit and preterm caregivers at home, together with a contact by phone, was shown to reduce anxiety amongst caregivers and to improve their ability to provide appropriate care [29]. A Neonatal Transitional Care Program in the United States that involved 10 to 20 follow-up home visits by HCWs over four months found reduced stress levels for caregivers, more successful breastfeeding routines and a reduction in the number of visits to hospital emergency departments [64]. Education and support groups commenced before discharge have also proved beneficial in aiding the transition from hospital to home, providing information to parents and opportunities for discussions with peers sharing similar experiences [30, 63, 65].

### Strengths and limitations

Purposive sampling methods used to achieve the study population contribute to the validity of the research [66]. However, in sampling, selection bias may have been introduced. We were unable to access many primary caregivers who did not attend follow-up appointments and who may have been facing greater challenges at home. Therefore, it would have been valuable to have followed up more caregivers by travelling to their homes. To mitigate this, caregivers were also identified and recruited through patient logbooks and offered transport costs to be able to attend interviews. Many fathers were unable or unwilling to take part, thus limiting insights into perceived gender roles.

Caregivers may have reported practising the care that they knew they *should* be providing, rather than the care that they *were* providing. An attempt was made to mitigate this by visiting some caregivers at home where they felt more at ease. This also made it possible to get a sense of the home environment and establish what care was occurring.

Due to the number of languages spoken in the area, interviews were often not conducted in the participants’ first language. This may also have had an impact on the quality of the results obtained.

Although the results of this research might not be generalisable to other settings due to differences in culture, beliefs and resources, the findings related to the care of preterm babies in the community in eastern Uganda have uncovered several important issues. These findings could, therefore, be used to generate hypotheses as a basis for future research in other low-resource settings.

## Conclusion

There was considerable good practice found in this NNU in eastern Uganda. Caregivers were well prepared for discharge. Weaknesses were found in follow-up care and support in the community, which led to feelings of isolation. The burden of responsibilities in the home, preconceptions about gender roles and financial considerations all provided impediments to care. Lack of practical and financial support from fathers and other family members and lack of provision at local healthcare facilities mean that, following discharge, the solid foundations laid in NNU began to crumble.

Based on our study, provision for ongoing communication by phone with neonatal staff after discharge and the arrangement of follow-up visits with experienced staff in the local community could lead to better care. Education of spouses and family members prior to discharge has the potential to greatly enhance the practical and psychosocial support for caregivers of preterm babies in the home. After discharge, these preterm infants remain at high risk and a clear need for parental training in basic neonatal resuscitation prior to discharge was identified. This has the potential not only to reduce fatalities in the community but to also reduce maternal fear and stress. Huge change cannot be expected. However, these small steps could pave the way to improved community care.

## Supplementary Information

**Additional file 1.**

**Additional file 2.**

## Data Availability

The datasets used during the current study are available from the corresponding author on reasonable request.

## Abbreviations

FGD: Focus group discussion
HCW: Health care worker
IDI: In-depth interview
MRRH: Mbale Regional Referral Hospital
NNU: Neonatal Unit
WHO: World Health Organization

## References

[1] Beck S, Wojdyla D, Say L, Betran AP, Merialdi M, Requejo JH (2010). The worldwide incidence of preterm birth: a systematic review of maternal mortality and morbidity. Bull World Health Organ.

[2] Conclusions from the WHO multicenter study of serious infections in young infants. The WHO Young Infants Study Group (1999). Pediatr Infect Dis J.

[3] Okello F, Egiru E, Ikiror J, Acom L, Loe K, Olupot-Olupot P (2019). Reducing preterm mortality in eastern Uganda: the impact of introducing low-cost bubble CPAP on neonates < 1500 g. BMC Pediatr.

[4] Waiswa P, Nyanzi S, Namusoko-Kalungi S, Peterson S, Tomson G, Pariyo GW (2010). ’I never thought that this baby would survive; I thought that it would die any time’: perceptions and care for preterm babies in eastern Uganda. Trop Med Int Health.

[5] World Health Organization MoD (2012). The partnership for maternal, newborn & child health, save the Children. Born too soon: the global action report on preterm birth.

[6] Conde-Agudelo A, Diaz-Rossello JL (2016). Kangaroo mother care to reduce morbidity and mortality in low birthweight infants. Cochrane Database Syst Rev.

[7] Beal JA (2005). Evidence for best practices in the neonatal period. MCN Am J Matern Child Nurs.

[8] Group WHOIKS, Arya S, Naburi H, Kawaza K, Newton S, Anyabolu CH (2021). Immediate “Kangaroo Mother Care” and survival of infants with low Birth Weight. N Engl J Med.

[9] Horta BLVC (2013). Long-term effects of breastfeeding: a systematic review.

[10] Boyd CA, Quigley MA, Brocklehurst P (2007). Donor breast milk versus infant formula for preterm infants: systematic review and meta-analysis. Arch Dis Child Fetal Neonatal Ed.

[11] Cacho NT, Parker LA, Neu J (2017). Necrotizing Enterocolitis and human milk feeding: a systematic review. Clin Perinatol.

[12] Ganapathy V, Hay JW, Kim JH (2012). Costs of necrotizing enterocolitis and cost-effectiveness of exclusively human milk-based products in feeding extremely premature infants. Breastfeed Med.

[13] Patel AL, Johnson TJ, Engstrom JL, Fogg LF, Jegier BJ, Bigger HR (2013). Impact of early human milk on sepsis and health-care costs in very low birth weight infants. J Perinatol.

[14] Quigley M, Embleton ND, McGuire W (2018). Formula versus donor breast milk for feeding preterm or low birth weight infants. Cochrane Database Syst Rev.

[15] Ronnestad A, Abrahamsen TG, Medbo S, Reigstad H, Lossius K, Kaaresen PI (2005). Late-onset septicemia in a norwegian national cohort of extremely premature infants receiving very early full human milk feeding. Pediatrics.

[16] Polin RA, Denson S, Brady MT (2012). Committee on F, Newborn, Committee on Infectious D. Strategies for prevention of health care-associated infections in the NICU. Pediatrics.

[17] Harbarth S, Pittet D, Grady L, Zawacki A, Potter-Bynoe G, Samore MH (2002). Interventional study to evaluate the impact of an alcohol-based hand gel in improving hand hygiene compliance. Pediatr Infect Dis J.

[18] Abdallah Y, Namiiro F, Nankunda J, Mugalu J, Vaucher Y (2018). Mortality among very low birth weight infants after hospital discharge in a low resource setting. BMC Pediatr.

[19] Leonard A, Mayers P (2008). Parents’ lived experience of providing kangaroo care to their preterm infants. Health SA Gesondheid.

[20] Hunter EC, Callaghan-Koru JA, Al Mahmud A, Shah R, Farzin A, Cristofalo EA (2014). Newborn care practices in rural Bangladesh: implications for the adaptation of kangaroo mother care for community-based interventions. Soc Sci Med.

[21] Sankey JJ, Brennan S (2001). Living with difference: caring for a premature baby at home. Collegian.

[22] Jackson K, Ternestedt BM, Schollin J (2003). From alienation to familiarity: experiences of mothers and fathers of preterm infants. J Adv Nurs.

[23] de Souza NL, Pinheiro-Fernandes AC, Clara-Costa Ido C, Cruz-Enders B, de Carvalho JB, da Silva Mde L (2010). Domestic maternal experience with preterm newborn children. Rev Salud Publica (Bogota).

[24] Phillips-Pula L, Pickler R, McGrath JM, Brown LF, Dusing SC (2013). Caring for a preterm infant at home: a mother’s perspective. J Perinat Neonatal Nurs.

[25] Reyna BA, Pickler RH, Thompson A (2006). A descriptive study of mothers’ experiences feeding their preterm infants after discharge. Adv Neonatal Care.

[26] Bazzano A, Hill Z, Tawiah-Agyemang C, Manu A, Ten Asbroek G, Kirkwood B (2012). Introducing home based skin-to-skin care for low birth weight newborns: a pilot approach to education and counseling in Ghana. Glob Health Promot.

[27] Arzani A, Valizadeh L, Zamanzadeh V, Mohammadi E (2015). Mothers’ strategies in handling the prematurely born infant: a qualitative study. J Caring Sci.

[28] Lingam R, Gupta P, Zafar S, Hill Z, Yousafzai A, Iyengar S (2014). Understanding care and feeding practices: building blocks for a sustainable intervention in India and Pakistan. Ann N Y Acad Sci.

[29] Birgitta Lindberg KA, Kerstin Öhrling (2009). Taking care of their baby at home but with nursing staff as support: the use of videoconferencing in providing neonatal support to parents of preterm infants. J Neonatal Nurs.

[30] Broedsgaard A, Wagner L (2005). How to facilitate parents and their premature infant for the transition home. Int Nurs Rev.

[31] Hemati Z, Namnabati M, Taleghani F, Sadeghnia A (2017). Mothers’ challenges after Infants’ discharge from neonatal intensive care unit: a qualitative study. Iran J Neonatol.

[32] Jefferies AL, Canadian Paediatric Society F, Newborn C (2014). Going home: facilitating discharge of the preterm infant. Paediatr Child Health.

[33] Lindberg B, Axelsson K, Öhrling K (2009). Taking care of their baby at home but with nursing staff as support: the use of videoconferencing in providing neonatal support to parents of preterm infants. J Neonatal Nurs.

[34] Ekirapa-Kiracho E, Namazzi G, Tetui M, Mutebi A, Waiswa P, Oo H (2016). Unlocking community capabilities for improving maternal and newborn health: participatory action research to improve birth preparedness, health facility access, and newborn care in rural Uganda. BMC Health Serv Res.

[35] Mrisho M, Schellenberg JA, Mushi AK, Obrist B, Mshinda H, Tanner M (2008). Understanding home-based neonatal care practice in rural southern Tanzania. Trans R Soc Trop Med Hyg.

[36] Quasem I, Sloan NL, Chowdhury A, Ahmed S, Winikoff B, Chowdhury AM (2003). Adaptation of kangaroo mother care for community-based application. J Perinatol.

[37] Mays N, Pope C (2000). Qualitative research in health care. Assessing quality in qualitative research. BMJ.

[38] UBoSUa I (2017). Uganda Demographic and Health Survey 2016: Key Indicators Report. UBOS, and Rockville.

[39] Burgoine K, Ikiror J, Akol S, Kakai M, Talyewoya S, Sande A (2018). Staged implementation of a two-tiered hospital-based neonatal care package in a resource-limited setting in Eastern Uganda. BMJ Glob Health.

[40] Rithchie J, Lewis J. Qualitative Research Practice: A Guide for Social Science Students and Researchers. First edition. SAGE; 2003.

[41] Gale NK, Heath G, Cameron E, Rashid S, Redwood S (2013). Using the framework method for the analysis of qualitative data in multi-disciplinary health research. BMC Med Res Methodol.

[42] Payne G, Payne J (2004). Key concepts in social research.

[43] Ritchie JaL J (2003). Qualitative research practice: a guide for social science students and researchers.

[44] Finch HaL J (2003). Qualitative research practice: a guide for social science students and researchers.

[45] McMaster P, Haina T, Vince JD (2000). Kangaroo care in Port Moresby, Papua New Guinea. Trop Doct.

[46] Ramanathan K, Paul VK, Deorari AK, Taneja U, George G (2001). Kangaroo Mother Care in very low birth weight infants. Indian J Pediatr.

[47] Parsons S, Mackinnon RJ (2009). Teaching parents infant resuscitation. Infant.

[48] Tawiah-Agyemang C, Kirkwood BR, Edmond K, Bazzano A, Hill Z (2008). Early initiation of breast-feeding in Ghana: barriers and facilitators. J Perinatol.

[49] Kesterton AJ, Cleland J (2009). Neonatal care in rural Karnataka: healthy and harmful practices, the potential for change. BMC Pregnancy Childbirth.

[50] Seidman G, Unnikrishnan S, Kenny E, Myslinski S, Cairns-Smith S, Mulligan B (2015). Barriers and enablers of kangaroo mother care practice: a systematic review. PLoS One.

[51] Chan GJ, Labar AS, Wall S, Atun R (2016). Kangaroo mother care: a systematic review of barriers and enablers. Bull World Health Organ.

[52] Charpak N, Tessier R, Ruiz JG, Hernandez JT, Uriza F, Villegas J (2017). Twenty-year follow-up of kangaroo mother care versus traditional care. Pediatrics.

[53] Kambarami RA, Mutambirwa J, Maramba PP (2002). Caregivers’ perceptions and experiences of ‘kangaroo care’ in a developing country. Trop Doct.

[54] Moxon SG, Lawn JE, Dickson KE, Simen-Kapeu A, Gupta G, Deorari A (2015). Inpatient care of small and sick newborns: a multi-country analysis of health system bottlenecks and potential solutions. BMC Pregnancy Childbirth.

[55] Nguah SB, Wobil PN, Obeng R, Yakubu A, Kerber KJ, Lawn JE (2011). Perception and practice of Kangaroo Mother Care after discharge from hospital in Kumasi, Ghana: a longitudinal study. BMC Pregnancy Childbirth.

[56] Blomqvist YT, Frolund L, Rubertsson C, Nyqvist KH (2013). Provision of Kangaroo Mother Care: supportive factors and barriers perceived by parents. Scand J Caring Sci.

[57] Flacking R, Ewald U, Nyqvist KH, Starrin B (2006). Trustful bonds: a key to “becoming a mother” and to reciprocal breastfeeding. Stories of mothers of very preterm infants at a neonatal unit. Soc Sci Med.

[58] Hong TM, Callister LC, Schwartz R (2003). First time mothers’ views of breastfeeding support from nurses. MCN Am J Matern Child Nurs.

[59] Wooldridge J, Hall WA (2003). Posthospitalization breastfeeding patterns of moderately preterm infants. J Perinat Neonatal Nurs.

[60] Lima G, Quintero-Romero S, Cattaneo A (2000). Feasibility, acceptability and cost of kangaroo mother care in Recife, Brazil. Ann Trop Paediatr.

[61] Kymre IG, Bondas T (2013). Balancing preterm infants’ developmental needs with parents’ readiness for skin-to-skin care: a phenomenological study. Int J Qual Stud Health Well-being.

[62] Blencowe H, Kerac M, Molyneux E (2009). Safety, effectiveness and barriers to follow-up using an ‘early discharge’ Kangaroo Care policy in a resource poor setting. J Trop Pediatr.

[63] Lopez GL, Anderson KH, Feutchinger J (2012). Transition of premature infants from hospital to home life. Neonatal Netw.

[64] Lasby K, Newton S, von Platen A (2004). Neonatal transitional care. Can Nurse.

[65] Melnyk BM, Feinstein NF, Alpert-Gillis L, Fairbanks E, Crean HF, Sinkin RA (2006). Reducing premature infants’ length of stay and improving parents’ mental health outcomes with the creating Opportunities for parent empowerment (COPE) neonatal intensive care unit program: a randomized, controlled trial. Pediatrics.

[66] Palinkas LA, Horwitz SM, Green CA, Wisdom JP, Duan N, Hoagwood K (2015). Purposeful sampling for qualitative data Collection and analysis in mixed method implementation research. Adm Policy Ment Health.

