# Supplementary material for: ‘They said, let’s teach you how you are going to care for the child at home…’: caregivers’ and healthcare worker’s perceptions and experiences of post-discharge preterm care in eastern Uganda
Source: BMC Health Serv Res. 2022 Dec 14;22:1521. doi: 10.1186/s12913-022-08894-3 (PMC9749343; doi:10.1186/s12913-022-08894-3)
Supplement: Supplementary file 1 — Additional file 1. [file 12913_2022_8894_MOESM1_ESM.docx]

**Supplementary material**

**Topic Guide IDI with Carer/Case-Study initial IDI**

*Leaving Hospital*

- Tell me about leaving the hospital
- Tell me about how you were prepared you were to care for your preterm at discharge
  - Probe: Tell me what worked?
  - Probe: What could have been better?
- How has your baby been since leaving the hospital?
  - Probe: Health – has baby been unwell? Tell me more about this
- Tell me about any problems you experienced after leaving the hospital
  - Subsections
    - Kangaroo Mother Care
    - Feeding
    - Sickness
  - Can you give us an example (of each mentioned).

*Caring at Home*

- Tell me about your first week at home
- Who was the primary carer while the baby was on the NNU?
  - Probe: Why?
- Who is the primary carer for your baby now that you are at home?
  - Probe: Why?
  - If Different: Is the carer who was at the hospital with the baby still around?
- Tell me about the transition from being in hospital to caring for your baby at home?
  - Probes: If struggled: tell me more/why?
  - Aspects of struggling:
    - Financial
    - Lack of Knowledge
    - Support
    - Culture
    - Did you have moral support from family/community/friends?
- What does Kangaroo Mother Care involve?
  - Why do you think you are asked to tie your baby to your chest?
  - Practically: How do you do it?
- Have you continued Kangaroo Mother Care at home?
  - Probe: How often? Who?
  - If no: Tell me more about this…Why? When did you stop?
  - What activities do you do whilst doing kangaroo care?
  - Do you do it at night?
- What other care does your baby receive at home?
  - Multivitamins/Sunshine Therapy/Iron/Aminophylline (if under 1.5Kg): How often? From who?
  - If yes: Why are you giving that medication?
  - If not, why not?
- Preterm babies are at high-risk of infection. What were you doing on the neonatal unit to prevent infection?
  - What are you doing at home to prevent infection?
- What has been the most difficult aspect of caring for your baby?
- What has helped you care for your baby?
  - Probe: For each answer – who/what has helped you?
- If your baby became very unwell, what would you do?
  - Who makes the decision?
  - Where would you go?
  - If you were given the choice – would you go to NNU/local healthcare facility?
- Do you know any other caregivers of preterms who had problems when they left the hospital caring for their baby?
  - Probe: What did they find difficult?
  - Probe: What do you think would have helped them?

*Future*

- Do you think you were prepared for discharge?
- Do you think you had enough knowledge for discharge?
- What could improve your ability to care for baby at home?
  - What would give you more confidence in caring?
  - How could we have better prepared you for discharge from the neonatal staff?
- If you had another preterm what would you do differently?
  - What would you like the staff to do differently?

**Topic Guide Follow-up IDI with Case-Studies**

*We had an interview about this.. I am now following up on how you have been*

- Tell me about your week with your baby/ time with your baby since we last met
- Now your baby is a bit older, tell me about a typical day with your baby now
  - How has it changed
  - Probe experiences
- How has the health of your baby been in the last week?
  - If unwell – can you explain how unwell the baby has been?
    - How was it managed?
- Is x still the primary carer?
  - If changed: Why?
  - Has this changed affected the care that has been given to the baby?
  - Who helps you care for your baby? What do they do?
  - If no-one: why noy?
- Tell me about any problems you have experienced this week related to taking care given to your baby
  - - Social
    - Psychosocial
    - Culture
    - Family
- Tell me about how kangaroo has gone this week/since we last met
  - Tell me when you do it
  - Tell me about how its worked
  - How many people are providing kangaroo care?
  - If no: When did you stop kangaroo care on this baby and why?
  - What have been the challenges?
- Why do you not do other chores when doing kangaroo?
  - What did the staff at the hospital tell you to do when doing kangaroo?
- What other care has your baby received at home in order to maintain his well-being?
  - Medication
  - Sun-therapy
- Tell me about how you have been maintaining the baby’s temperature since we last spoke?
- Tell me about how you have been maintaining the hygiene of the baby since we last spoke?
- What helps you do what you need to do for your baby?
- What stands in the way of you being able to care for your baby?
- If your baby stopped breathing what would you do?
  - Probe their knowledge
    - where did they learn what to do?
    - How would they do it in practice – can you show me?
  - Would it have been helpful to have been taught basic life support while at the hospital before discharge?
  - What would it have been helpful to know?
- Tell me about how your mood has been this week
  - Have you felt happy/sad at all?
  - Probe further: why? How did you manage it?

(Home Visits) Can you show me

- Where the baby sleeps
  - Is this where you keep the baby from other visitors?
- Can you show me where you do kangaroo?
- Can you show me where you and you visitors wash your hands
- Where you clean the utensils for feeding
- Where you do the sun bathing

**Supplement Topic Guide for FGDs with Caregivers Post-Discharge**

*Kangaroo Mother Care*

- What does Kangaroo Mother Care involve?
  - Why do you think you are asked to tie your baby to your chest?
  - Practically: How do you do it?
- Have you continued Kangaroo Mother Care at home?
  - Probe: How often? Who?
  - If no: Tell me more about this…Why? When did you stop?
  - What activities do you do whilst doing kangaroo care?
  - Do you do it at night?

*Other aspects of care*

- What other care does your baby receive at home?
  - Multivitamins/Sunshine Therapy/Iron/Aminophylline (if under 1.5Kg): How often? From who?
  - If yes: Why are you giving that medication?
  - If not, why not?
- Preterm babies are at high-risk of infection. What were you doing on the neonatal unit to prevent infection?
  - What are you doing at home to prevent infection?

*Caring at Home*

- Tell me about leaving the hospital
  - Probe: What guidance were you given on caring for your preterm at home?
  - Probe: What helped prepare you for baring for your preterm at home?
  - What could we have done better to prepare you for caring for your preterm at home?
- Tell me about the transition from being in hospital to caring for your baby at home?
  - Probes: If struggled: tell me more/why?
  - Aspects of struggling:
    - Financial
    - Lack of Knowledge
    - Support
    - Culture\
- Tell me about any problems you experienced after leaving the hospital
  - Subsections
    - Kangaroo Mother Care
    - Feeding
    - Sickness
  - Can you give us an example (of each mentioned).
- What has been the most difficult aspect of caring for your baby?
- What has helped you care for your baby?
  - Probe: For each answer – who/what has helped you?
    - Did you have moral support from family/community/friends?
- If your baby became very unwell, what would you do?
  - Who makes the decision?
  - Where would you go?
  - If you were given the choice – would you go to NNU/local healthcare facility?

*Future*

- Do you think you were prepared for discharge?
- Do you think you had enough knowledge for discharge?
- What could improve your ability to care for baby at home?
  - What would give you more confidence in caring?
  - How could we have better prepared you for discharge from the neonatal staff?
- If you had another preterm what would you do differently?
  - What would you like the staff to do differently?

**Topic Guide for FGD with Caregivers on Ward Pre-Discharge**

*Intro*

- How long have you been on the NNU?
- What is your relationship to the baby?
  - If not mother
    - Why?
    - Where is mother?
    - Who will be the primary carer of the baby once discharged?

*Preparing for home*

- How are you being prepared to go home and care for your baby?
  - What have the staff taught you?
    - If not the mother – how will the mother be taught about these things?
  - Do you feel ready to go home?
- How do you feel about going home?
  - Is there anything you are worried about?
  - Is there anything you think you will struggle with at home?
    - Finances
    - Support
    - Knowledge
    - Culture
- What do you think will help you caring for your baby once you have left the hospital?
  - How could the staff better prepare you to go home while you are on the ward?

*Kangaroo Mother Care*

- What does Kangaroo Mother Care involve?
  - Why do you think you are asked to tie your baby to your chest?
  - Practically: How do you do it?
- Tell me about how you will continue Kangaroo mother care at home?
  - Probe: How often?
  - Who?
  - When?
  - When will you stop?
  - What activities will you do whilst doing kangaroo care?
  - What do you think will be difficult when doing kangaroo at home?

*Other aspects of care*

- What other care will your baby receive at home?
  - Multivitamins
  - Sunshine Therapy
  - Aminophylline (if under 1.5Kg): How often? From who?
  - Why are you giving that medication?
- Preterm babies are at high-risk of infection. How will you prevent infection at home?
  - What do you think might be hard?
  - What will help you?
- How will you monitor the baby’s temperature when you are at home?
  - What do you think might be hard?
  - What will help you?

**Supplement 2: Topic Guide IDIs with Heatlhcare Workers**

*Leaving Hospital*

- What care should caregivers of preterms be continuing at home?
- How are caregivers of preterms prepared to take care of their baby at home before being discharged?

*Caring at Home*

- Do you believe caregivers are able to continue a good quality of care once discharged into the community?
  - Probe: Tell me about what you think works
  - Probe: Tell me about what you think doesn’t work
- What assistance is given to caregivers once they have left the hospital?
- Have caregivers ever mentioned they have been struggling after discharge when at follow-up appointments/on returning to the hospital?
  - Probe: Can you think of some examples? Tell me about these? What happened in these cases?
- In your opinion, what are the barriers facing caregivers in continuing care for their preterm babies after being discharged?
- In your opinion, what improves the ability of caregivers to care for their preterm babies in the community?

*Future*

- What could improve the care of babies following discharge?
- What do you think some staff do well
- What do you think some could do better?
- If you had funding to change something, what would it be?
- What training needs for medical workers?
- How do you think we could better prepare caregivers for discharge?
