# Supplementary material for: ‘They said, let’s teach you how you are going to care for the child at home…’: caregivers’ and healthcare worker’s perceptions and experiences of post-discharge preterm care in eastern Uganda
Source: BMC Health Serv Res. 2022 Dec 14;22:1521. doi: 10.1186/s12913-022-08894-3 (PMC9749343; doi:10.1186/s12913-022-08894-3)
Supplement: Supplementary file 2 — Additional file 2. [file 12913_2022_8894_MOESM2_ESM.docx]

**Supplement 2: Patient demographics**

| **Caregivers Participant Code** | **Relationship to Preterm** | **Age** | **No. of Children** |
| --- | --- | --- | --- |
| Case Studies | | | |
| CS.M.1 | Mother | 20-30 | 1 |
| CS.M.2 | Mother | 20-30 | 2 |
| CS.M.3 | Mother | 30-40 | 2 |
| CS.GM.1 | Grandmother | 50+ | Unknown |
| In-Depth Interviews | | | |
| IDI.F.1 | Father | 20-30 | 2 |
| IDI.M.1 | Mother | 30-40 | 4 |
| IDI.M.2 | Mother | 20-30 | 2 |
| IDI.M.3 | Mother | 20-30 | 3 |
| IDI.M.4 | Mother | 30-40 | 3 |
| IDI.M.5 | Mother | 20-30 | 4 |
| IDI.M.6 | Mother | 30-40 | 5 |
| IDI.GM.1 | Grandmother | 40-50 | Unknown |
| IDI.M.7 | Mother | Unknown | Unknown |
| IDI.GM.2 | Grandmother | 40-50 | Unknown |
| IDI.M.8 | Mother | Unknown | Unknown |
| IDI.GM.3 | Grandmother | 50+ | Unknown |
| Focus Group Discussion 1 | | | |
| FGD.M.1 | Mother | 30-40 | 4 |
| FGD.M.2 | Mother | 30-40 | 7 |
| FGD.M.3 | Mother | 20-30 | 1 |
| FGD.A.1 | Aunt | <20 | 0 |
| Focus Group Discussion 2 | | | |
| FGD.M.4 | Mother | Unknown | Unknown |
| FGD.M.5 | Mother | Unknown | Unknown |
| FGD.M.6 | Mother | Unknown | Unknown |
| FGD.M.7 | Mother | 20-30 | 2 |
| FGD.M.8 | Mother | 20-30 | 1 |
| FGD.F.1 | Father | 20-30 | 1 |
| FGD.GM.1 | Grandmother | 40-50 | Unknown |
| Focus Group Discussion 3 | | | |
| FGD.M.9 | Mother | 20-30 | 2 |
| FGD.M.10 | Mother | 20-30 | 3 |
| FGD.GM.2 | Grandmother | 40-50 | Unknown |
| FGD.GM.3 | Grandmother | 40-50 | Unknown |
| FGD.M.11 | Mother | 20-30 | 2 |
| FGD.A.2 | Aunt | 20-30 | 4 |

| **HCWs Participant Code** | **Role at MRRH** |
| --- | --- |
| IDI.HCW1.1 | Lead Nurse on NNU |
| IDI.HCW1.2 | Antenatal Clinic Nurse – Previously worked on NNU |
| IDI.HCW1.3 | Nurse working on NNU |
| IDI.HCW1.4 | Nurse working on NNU |
| IDI.HCW2.1 | Doctor on NNU |
| IDI.HCW3.1 | Health Educator on NNU |
